# Supplementary material for: Appraising the causal relevance of DNA methylation for risk of lung cancer
Source: Int J Epidemiol. 2019 Sep 24;48(5):1493–504. doi: 10.1093/ije/dyz190 (PMC6857764; doi:10.1093/ije/dyz190)
Supplement: dyz190_Supplementary_Data [file dyz190_supplementary_data.docx]

# Supplementary Material

# Appraising the causal relevance of DNA methylation for risk of lung cancer

Thomas Battram^1,2λ*^, Rebecca C Richmond^1,2λ^, Laura Baglietto^3†^, Philip C Haycock^1,2†^, Vittorio Perduca^4^, Stig E Bojesen^5,6,7^, Tom R Gaunt^1,2^, Gibran Hemani^1,2^, Florence Guida^8^, Robert Carreras-Torres^8^, Rayjean Hung^9^, Christopher I Amos^10^, Joshua R Freeman^11^, Torkjel M Sandanger^12^, Therese H Nøst^13^, Børge Nordestgaard^5,6,7^, Andrew E Teschendorff^14,15,16^, Silvia Polidoro^17^, Paolo Vineis^17,18^, Gianluca Severi^19,20,21,22^, Allison M Hodge^21,22^, Graham G Giles^21,22^, Kjell Grankvist^23^, Mikael B Johansson^24^, Mattias Johansson^8^, George Davey Smith^1,2$^, Caroline L Relton^1,2$^

# **Supplementary Methods**

## **EWAS studies**

At the various laboratory sites, samples were distributed into 96-well plates and processed in chips of 12 arrays (8 chips per plate) with case-control pairs arranged randomly on the same chip. Methylation data were pre-processed and normalized in each study, and probe filtering was performed as previously described (1), leaving 465,886 CpGs suitable for the analysis in EPIC-Italy, 485,330 CpGs in MCCS, 450,890 CpGs in NOWAC and 482,867 CpGs in NSHDS.

### European Prospective Investigation into Cancer and Nutrition-Italy (EPIC-Italy)

EPIC-Italy includes 47,749 volunteers (32,579 women) aged 35–70 years at the time of recruitment (1992–1998). Anthropometric measurements and lifestyle variables including detailed information on smoking history were collected at recruitment through standardized questionnaires, together with a blood sample. Within EPIC-Italy we conducted a nested case-control study utilizing incident cases diagnosed within follow-up and healthy controls individually matched to cases by gender, date of birth (±5 years), date of inclusion in the study and study centre. Analysis was performed for 185 incident cases diagnosed within follow-up and matched controls. Laboratory procedures were carried out at the Human Genetics Foundation (Turin, Italy) and DNA extracted from buffy coats as previously described (1). All participants signed an informed consent form, and the ethical review boards of the International Agency for Research on Cancer and of each local participating centre approved the study protocol.

### Melbourne Collaborative Cohort Study (MCCS)

The MCCS is a prospective cohort study of 41,514 volunteers (24,469 women) aged between 27 and 76 years at baseline (1990-1994). At baseline attendance, participants completed questionnaires that measured demographic characteristics and lifestyle factors. Height and weight were directly measured, and a blood sample was collected and stored. Incident cases of lung cancer were identified through linkage with the State and National Cancer Registries during follow-up up to the end of 2011. The MCCS sample included 367 cases and 367 matched controls selected from MCCS participants who were lung cancer free at the age of diagnosis of the matching case (density sampling). Matching variables included gender, date of blood collection (within 6 months), date of birth (within 1 year), country of birth (Australia and UK versus Southern Europe), type of biospecimen (lymphocyte, buffy coat and dried blood spot) and smoking status (never smokers; short-term former smokers: quitting smoking less than 10 years before blood draw; long-term former smokers: quitting smoking 10 years or more before blood draw; current light smokers: less than 15 cigarettes per day at blood draw; and current heavy smokers: 15 cigarettes or more at blood draw). For the MCCS, laboratory procedures were carried out at the Genetic Epidemiology Laboratory, the University of Melbourne according to manufacturers' protocols. DNA extraction from lymphocytes and buffy coats was performed as previously described (1). The Cancer Council Victoria's Human Research Ethics Committee approved the study protocol. Subjects gave written consent to participate and for the investigators to obtain access to their medical records.

### Norwegian Women and Cancer (NOWAC)

The biobank of the NOWAC cohort was established in the years 2003-2006. Those who filled in an eight-page questionnaire and accepted the invitation to donate blood were sent blood drawing equipment together with a two-page epidemiological questionnaire. Around 50 000 women returned two tubes of blood to the Institute of Community Medicine at UiT The Arctic University of Norway and data linkage to the National Cancer Registry of Norway was performed. During follow-up to the end of 2011, 132 eligible cases of lung cancer were identified and were used for the EWAS. For each case, one control with an available blood sample was selected and matched on time since blood sampling and year of birth in order to control for effects of storage time and ageing. The cases and the controls were processed together for all laboratory procedures in order to reduce any batch effect. Laboratory procedures were carried out at the Human Genetics Foundation (Turin, Italy). DNA extraction from buffy coats was performed as previously described (1). All participants gave informed consent. The study was approved by the Regional Committee for Medical and Health Research Ethics in North Norway. Data storage and linkage was approved by the Norwegian Data Inspectorate.

### Northern Sweden Health and Disease Study (NSHDS)

NSHDS is an ongoing prospective cohort and intervention study intended for health promotion of the population of Västerbotten County in northern Sweden. All residents were invited to participate by attending a health check-up at their local health care centre at 40, 50 and 60 years of age. At the health check-up, participants were asked to complete a self-administered questionnaire covering various factors such as education, smoking habits, physical activity and diet. In addition, height and weight were measured and participants were asked to donate a blood sample. Incident lung cancer cases were identified through linkage to the regional cancer registry. One control was chosen at random for each lung cancer case from appropriate risk sets consisting of all cohort members alive and free of cancer (except non-melanoma skin cancer) at the time of diagnosis of the index case. Matching criteria were the same as for the MCCS except there was no matching for type of biospecimens as DNA was extracted from whole blood for all samples. After quality control, a total of 234 incident lung cancer cases and 234 individually matched controls were available for this analysis. Laboratory procedures for NSHDS were carried out at two sites. DNA extraction from the buffy coat was conducted at Umeå University, Sweden, as previously described. Illumina Infinium HumanMethylation450 BeadChip analysis was conducted at the ALSPAC/IEU Laboratory at the University of Bristol. All study subjects provided written informed consent at time of the recruitment into the NSHDS.

## **Mendelian randomization analysis**

### Accessible Resource for Integrated Epigenomic Studies (ARIES)

#### DNA methylation

Samples were drawn from the Avon Longitudinal Study of Parents and Children (2, 3). Blood from 1,018 mother–child pairs were selected for analysis as part of the Accessible Resource for Integrative Epigenomic Studies (ARIES, <http://www.ariesepigenomics.org.uk/>) (4). There are three timepoints in children and two in their mothers, the timepoints with mean ages (in brackets) are as follows in ARIES are as follows for children: birth, childhood (7.5), adolescence (17.1) and for mothers: during pregnancy (28.7), and at middle age (46.9). Following DNA extraction, samples were bisulphite converted using the Zymo EZ DNA Methylation™ kit (Zymo, Irvine, CA, USA). Following conversion, genome-wide methylation was measured using the Illumina Infinium HumanMethylation450 (HM450) BeadChip. Methylation data were normalised in R with the watermelon package (5) using the Touleimat and Tost (6) algorithm to reduce the non-biological differences between probes. Methylation data in ARIES were rank-normalised to remove outliers, and then Matrix eQTL software (7) was used to perform preliminary association analysis of SNPs with all CpG sites in the Illumina Infinium HM450 array with the exception of those failing QC, and those reported to map to more than one location (n=19,834) or to contain a genetic variant at the CpG site (n=74,182) (8).

#### Genetic data

Children were genotyped using the Illumina HumanHap550 quad genome-wide SNP genotyping platform (Illumina Inc., San Diego, USA) by the Wellcome Trust Sanger Institute (WTSI, Cambridge, UK) and the Laboratory Corporation of America (LCA, Burlington, NC, USA). Individuals were excluded on the basis of incorrect gender assignment; abnormal heterozygosity (<0.320 or >0.345 for WTSI data; <0.310 or >0.330 for LCA data); high missingness (>3%); cryptic relatedness (>10% identity by descent) and non-European ancestry (detected by multidimensional scaling analysis). Following QC the final directly genotyped dataset contained 500,527 SNP loci.

Mothers were genotyped using the Illumina Human660W-quad genome-wide SNP genotyping platform (Illumina Inc., San Diego, USA) at the Centre National de Génotypage (CNG, Paris, France). Individuals were excluded based on non-European ancestry, missingness, relatedness, gender mismatches and heterozygosity. PLINK (v1.07) (9) was used to carry out quality control measures on an initial set of 10,015 subjects and 557,124 directly genotyped SNPs. Following QC the final directly genotyped dataset contained 526,688 SNP loci.

Imputation was performed to increase the SNP density for all genotyped mothers and children combined. Genotypes were phased together using ShapeIt, and then imputed against the 1000 genomes reference panel (phase 1 version 3, phased using ShapeIt version 2, December 2013, using all populations) using Impute (version 2.2.2). Genotypes were filtered to have Hardy-Weinberg equilibrium P > 5x10~10-7, MAF > 1% and imputation info score > 0.8. Best guess genotypes were used for subsequent analysis. The final imputed dataset used for the analyses presented here contained 8,074,398 loci.

Written informed consent has been obtained from all ALSPAC participants. Ethical approval for the study was obtained from the ALSPAC Ethics and Law Committee and Local Research Ethics Committees.

Please note that the study website contains details of all the data that is available through a fully searchable data dictionary: <http://www.bris.ac.uk/alspac/researchers/data-access/data-dictionary/>.

### Northern Sweden Health and Disease Study (NSHDS)

The same NSHDS samples on which DNA methylation was measured were genotyped using the Illumina Infinium OncoArray-500k BeadChip (Illumina Inc. San Diego, CA) and quality control parameters were applied under the recently published TRICL-ILCCO GWAS study on lung cancer (10). Genetic imputation was performed on these samples using the Haplotype Reference Consortium (HRC) Panel (release 1) (11) through the Michigan Imputation Server (12).

### Copenhagen City Heart Study (CCHS)

Copenhagen City Heart Study is a prospective study of the general population (13). Copenhagen residents were invited to complete a questionnaire and undergo a physical examination and are followed through a unique person identifier in the Danish health registries. All participants gave written informed consent, and a Danish ethics committee approved the study (KF100.2039/91).

#### Phenotypic data

Participants were asked whether they smoked at the day of attendance or previously. If they answered affirmative to either of these questions, they were asked about their current and former smoking behaviour, including age of smoking initiation, age of smoking cessation, and number of daily consumed cigarettes, cheroots, cigars, and weekly grams of pipe tobacco. Based on these answers, participants were categorized as never, former, and current smokers. In addition, participants reported on alcohol consumption, occupational exposure to dust and/or welding fumes, exposure to passive smoking, education, and familial cases of lung cancer. The answers were reviewed together with an examiner at the day of attendance. Body mass index was calculated as measured weight in kilograms divided by measured height (in meters) squared.

#### Methylation data

At the physical examination, blood samples were drawn for DNA from which *AHRR* methylation extent was measured (14). The *AHRR* cg05575921 methylation extent was measured in duplicate samples of bisulphite treated DNA from peripheral blood from 9,234 individuals. We used a Taqman assay developed in our own laboratory, and included standard curves, as well as internal controls in each 384-well plate. Coefficients of variation at the methylation level of 71% varied from 5.0 to 6.7%. Laboratory technicians were blinded to smoking and disease status of the individuals. Results were validated with pyrosequencing on a subset of samples.

#### Genetic data

Genotypes from the iCOGs array (15) and prospective data on lung cancer incidence were also available for these participants. Of the 9234 individuals, genotype data from iCOGS on 8778 were available. In short, DNA isolated from leukocytes was genotyped with a custom Illumina iSelect genotyping array, designed to test genetic variants related to breast, ovary and prostate cancer, comprising ~211,000 SNPs after rigorous quality control.(15)

#### Identification of mQTLs for CCHS one-sample MR

mQTLs located within 1Mb of cg05575921 *AHRR* were identified in ARIES (FDR<0.05). Of those mQTLs which replicated within the CCHS, we performed an LD pruning step using a less stringent r^2^ threshold of 0.2 and generated an unweighted allele score, calculated by coding and then summing the alleles to reflect the average number of methylation-increasing alleles carried by an individual.

#### Lung cancer data

For lung cancer (ICD7, codes 1624 or 4624 until 1977, and ICD10, code C34 from 1978 and onwards), the date of first diagnosis was taken from the national Danish Cancer Registry from 1943 to December 2012.

### Transdisciplinary Research in Cancer of the Lung and The International Lung Cancer Consortium (TRICL-ILCCO)

Summary-level SNP effect estimates for lung cancer were obtained from the TRICL-ILCCO consortium, which has conducted GWAS on lung cancer overall (29,863 cases, 55,586 controls) for individuals genotyped using the Illumina Infinium OncoArray-500K BeadChip (Illumina Inc. San Diego, CA) and independent samples for which prior genotyping was performed (10).

# References

1. Baglietto L, Ponzi E, Haycock P, Hodge A, Bianca Assumma M, Jung CH, et al. DNA methylation changes measured in pre-diagnostic peripheral blood samples are associated with smoking and lung cancer risk. Int J Cancer. 2017;140(1):50-61.

2. Boyd A, Golding J, Macleod J, Lawlor DA, Fraser A, Henderson J, et al. Cohort Profile: the 'children of the 90s'--the index offspring of the Avon Longitudinal Study of Parents and Children. Int J Epidemiol. 2013;42(1):111-27.

3. Fraser A, Macdonald-Wallis C, Tilling K, Boyd A, Golding J, Davey Smith G, et al. Cohort Profile: the Avon Longitudinal Study of Parents and Children: ALSPAC mothers cohort. Int J Epidemiol. 2013;42(1):97-110.

4. Relton CL, Gaunt T, McArdle W, Ho K, Duggirala A, Shihab H, et al. Data Resource Profile: Accessible Resource for Integrated Epigenomic Studies (ARIES). Int J Epidemiol. 2015;44(4):1181-90.

5. Pidsley R, CC YW, Volta M, Lunnon K, Mill J, Schalkwyk LC. A data-driven approach to preprocessing Illumina 450K methylation array data. BMC genomics. 2013;14:293.

6. Touleimat N, Tost J. Complete pipeline for Infinium((R)) Human Methylation 450K BeadChip data processing using subset quantile normalization for accurate DNA methylation estimation. Epigenomics. 2012;4(3):325-41.

7. Gaunt TR, Shihab HA, Hemani G, Min JL, Woodward G, Lyttleton O, et al. Systematic identification of genetic influences on methylation across the human life course. Genome Biol. 2016;17:61.

8. Naeem H, Wong NC, Chatterton Z, Hong MK, Pedersen JS, Corcoran NM, et al. Reducing the risk of false discovery enabling identification of biologically significant genome-wide methylation status using the HumanMethylation450 array. BMC genomics. 2014;15:51.

9. Purcell S, Neale B, Todd-Brown K, Thomas L, Ferreira MAR, Bender D, et al. PLINK: A tool set for whole-genome association and population-based linkage analyses. Am J Hum Genet. 2007;81(3):559-75.

10. McKay JD, Hung RJ, Han Y, Zong X, Carreras-Torres R, Christiani DC, et al. Large-scale association analysis identifies new lung cancer susceptibility loci and heterogeneity in genetic susceptibility across histological subtypes. Nat Genet. 2017;49(7):1126-32.

11. McCarthy S, Das S, Kretzschmar W, Delaneau O, Wood AR, Teumer A, et al. A reference panel of 64,976 haplotypes for genotype imputation. Nat Genet. 2016;48(10):1279-83.

12. Das S, Forer L, Schonherr S, Sidore C, Locke AE, Kwong A, et al. Next-generation genotype imputation service and methods. Nat Genet. 2016;48(10):1284-7.

13. Kaur-Knudsen D, Bojesen SE, Tybjaerg-Hansen A, Nordestgaard BG. Nicotinic acetylcholine receptor polymorphism, smoking behavior, and tobacco-related cancer and lung and cardiovascular diseases: a cohort study. J Clin Oncol. 2011;29(21):2875-82.

14. Bojesen SE, Timpson N, Relton C, Davey Smith G, Nordestgaard BG. AHRR (cg05575921) hypomethylation marks smoking behaviour, morbidity and mortality. Thorax. 2017.

15. Bojesen SE, Pooley KA, Johnatty SE, Beesley J, Michailidou K, Tyrer JP, et al. Multiple independent variants at the TERT locus are associated with telomere length and risks of breast and ovarian cancer. Nat Genet. 2013;45(4):371-84, 84e1-2.

**Table S1. Instrument strength in ARIES**

| **SNP** | **CpG** | **Beta** | **SE** | **P** | **N** | **F** | **R2** |
| --- | --- | --- | --- | --- | --- | --- | --- |
| rs1048691 | cg23387569 | 0.355 | 0.053 | 3.87E-11 | 834 | 44.87 | 0.05 |
| rs1939110 | cg11660018 | -0.404 | 0.048 | 2.61E-16 | 834 | 69.90 | 0.08 |
| rs13087163 | cg01901332 | -0.194 | 0.035 | 5.82E-08 | 834 | 29.97 | 0.03 |
| rs7927381 | cg01901332 | 0.382 | 0.069 | 3.94E-08 | 834 | 30.75 | 0.04 |
| rs878481 | cg05951221 | -0.319 | 0.042 | 5.93E-14 | 834 | 58.38 | 0.07 |
| rs1048691 | cg16823042 | 0.321 | 0.053 | 2.85E-09 | 834 | 36.06 | 0.04 |
| rs734568 | cg03636183 | 0.284 | 0.043 | 6.74E-11 | 834 | 43.73 | 0.05 |
| rs72967500 | cg23771366 | -0.628 | 0.062 | 1.27E-22 | 834 | 101.59 | 0.11 |
| rs3748971 | cg21566642 | -0.502 | 0.080 | 5.34E-10 | 834 | 39.48 | 0.05 |
| rs9643220 | cg25305703 | 0.343 | 0.049 | 7.09E-12 | 834 | 48.38 | 0.05 |
| rs77433148 | cg08709672 | -0.804 | 0.137 | 6.35E-09 | 834 | 34.44 | 0.04 |
| rs17518433 | cg09935388 | -0.330 | 0.046 | 1.80E-12 | 834 | 51.23 | 0.06 |
| rs463924 | cg26963277 | -0.394 | 0.045 | 6.80E-18 | 834 | 77.73 | 0.09 |
| rs56080708 | cg27241845 | 0.716 | 0.070 | 2.41E-23 | 834 | 105.29 | 0.11 |
| rs11744553 | cg05575921 | 0.217 | 0.040 | 7.22E-08 | 834 | 29.53 | 0.03 |
| rs11746538 | cg05575921 | -0.369 | 0.058 | 2.99E-10 | 834 | 40.67 | 0.05 |

SE = standard error

P = P value

N = sample size

F = F statistic

**Table S2. Heterogeneity between studies and smoker groups in the meta-analysis of EWAS in 4 cohorts**

|  |  |  |  | **Basic** | | | **SV adjusted** | | | **SV and cell count** | | | **Never smokers** | | | **Former smokers** | | | **Current smokers** | | |
| --- | --- | --- | --- | --- | --- | --- | --- | --- | --- | --- | --- | --- | --- | --- | --- | --- | --- | --- | --- | --- | --- |
| **CpG** | **Gene** | **Chr** | **Position** | **Dir** | **I2** | **P** | **Dir** | **I2** | **P** | **Dir** | **I2** | **P** | **Dir** | **I2** | **P** | **Dir** | **I2** | **P** | **Dir** | **I2** | **P** |
| cg01901332 | ARRB1 | 11 | 75031054 | ---- | 0 | 0.631 | ---- | 12 | 0.335 | ---- | 29 | 0.237 | +--- | 11 | 0.337 | ---- | 0 | 0.970 | ---- | 18 | 0.301 |
| cg01940273 | ALPPL2 | 2 | 233284934 | ---- | 35 | 0.201 | ---- | 61 | 0.053 | ---- | 60 | 0.056 | +--+ | 59 | 0.064 | ---- | 7 | 0.356 | ---+ | 34 | 0.206 |
| cg03636183 | F2RL3 | 19 | 17000585 | ---- | 42 | 0.159 | ---- | 76 | 0.006 | ---- | 71 | 0.015 | +-+- | 26 | 0.254 | ---- | 30 | 0.231 | ---- | 0 | 0.540 |
| cg05575921 | AHRR | 5 | 373378 | ---- | 47 | 0.131 | ---- | 73 | 0.012 | ---- | 70 | 0.018 | +--+ | 0 | 0.481 | ---- | 0 | 0.433 | ---- | 34 | 0.207 |
| cg05951221 | ALPPL2 | 2 | 233284402 | ---- | 45 | 0.139 | ---- | 61 | 0.053 | ---- | 54 | 0.092 | --++ | 0 | 0.862 | ---- | 33 | 0.217 | ---- | 15 | 0.315 |
| cg06126421 | IER3 | 6 | 30720080 | ?--- | 69 | 0.041 | ---- | 67 | 0.027 | ---- | 68 | 0.024 | ?--- | 0 | 0.464 | ?--- | 11 | 0.326 | ?--- | 0 | 0.400 |
| cg08709672 | AVPR1B | 1 | 206224334 | ---- | 12 | 0.333 | ---- | 52 | 0.101 | ---- | 57 | 0.071 | --++ | 55 | 0.085 | ---+ | 0 | 0.584 | ---+ | 0 | 0.735 |
| cg09935388 | GFI1 | 1 | 92947588 | ---- | 20 | 0.291 | ---- | 50 | 0.110 | ---- | 29 | 0.241 | ---+ | 0 | 0.967 | -+-- | 60 | 0.056 | ---- | 0 | 0.729 |
| cg11660018 | PRSS23 | 11 | 86510915 | ---- | 0 | 0.476 | ---- | 7 | 0.358 | ---- | 0 | 0.455 | +-++ | 0 | 0.699 | ---- | 9 | 0.349 | ---- | 0 | 0.557 |
| cg16823042 | AGAP2 | 12 | 58119992 | ---- | 13 | 0.330 | --+- | 0 | 0.482 | --+- | 0 | 0.554 | +-+- | 19 | 0.293 | ---+ | 68 | 0.024 | ---+ | 0 | 0.919 |
| cg21566642 | ALPPL2 | 2 | 233284661 | ---- | 46 | 0.136 | ---- | 38 | 0.184 | ---- | 18 | 0.300 | +-++ | 0 | 0.681 | ---- | 0 | 0.798 | ---- | 65 | 0.035 |
| cg23387569 | AGAP2 | 12 | 58120011 | ---- | 29 | 0.239 | --+- | 35 | 0.204 | --+- | 22 | 0.279 | --+- | 0 | 0.633 | ---+ | 76 | 0.005 | ---+ | 0 | 0.624 |
| cg23771366 | PRSS23 | 11 | 86510998 | ---- | 42 | 0.161 | ---- | 75 | 0.007 | ---- | 72 | 0.013 | ++++ | 0 | 0.805 | ---- | 0 | 0.518 | ---+ | 27 | 0.249 |
| cg25305703 | CASC21 | 8 | 128378218 | ---- | 53 | 0.096 | ---- | 0 | 0.461 | ---- | 4 | 0.373 | ---- | 0 | 0.780 | ---- | 28 | 0.246 | ---- | 0 | 0.793 |
| cg26963277 | KCNQ1 | 11 | 2722407 | ---- | 0 | 0.512 | ---- | 0 | 0.516 | ---- | 0 | 0.430 | --++ | 53 | 0.095 | ---+ | 0 | 0.466 | ---- | 17 | 0.308 |
| cg27241845 | ALPPL2 | 2 | 233250370 | ---- | 57 | 0.075 | ---- | 48 | 0.122 | ---- | 41 | 0.163 | ---+ | 0 | 0.643 | ---+ | 0 | 0.672 | ---- | 32 | 0.221 |

Dir = direction of effect

SV = surrogate variable

Smoker group comparison = heterogeneity between the meta-analysis effect estimates for never smokers, former smokers and current smokers

**Table S3. The SNP-exposure association estimates from ARIES and NSHDS**

| **CpG** | **CpG Chr** | **CpG Pos** | **Gene region** | **SNP** | **SNP Chr** | **SNP Pos** | **A1** | **A2** | **MAF** | **Beta (95% CI)** | **P** | **NSHDS Beta (95% CI)** | **NSHDS P** | **Trans** |
| --- | --- | --- | --- | --- | --- | --- | --- | --- | --- | --- | --- | --- | --- | --- |
| cg16823042 | 12 | 58119992 | AGAP2 | rs1048691 | 12 | 58152948 | T | C | 0.207 | 0.321 (0.216, 0.426) | 2.85E-09 | 0.176 (0.031, 0.322) | 0.018 | N |
| cg23387569 | 12 | 58120011 | AGAP2 | rs1048691 | 12 | 58152948 | T | C | 0.208 | 0.355 (0.251, 0.458) | 3.87E-11 | 0.186 (0.04, 0.331) | 0.012 | N |
| cg05575921* | 5 | 373378 | AHRR | rs11746538 | 5 | 427466 | A | C | 0.121 | -0.369 (-0.482, -0.255) | 2.99E-10 | -0.062 (-0.315, 0.19) | 0.629 | N |
| cg05575921* | 5 | 373378 | AHRR | rs11744553 | 5 | 26366 | C | G | 0.311 | 0.217 (0.139, 0.295) | 7.22E-08 | 0.085 (-0.058, 0.228) | 0.243 | N |
| cg27241845 | 2 | 233250370 | ALPPL2 | rs56080708 | 2 | 233274475 | A | C | 0.078 | 0.716 (0.579, 0.852) | 2.41E-23 | 0.464 (0.244, 0.684) | 3.61E-05 | N |
| cg05951221 | 2 | 233284402 | ALPPL2 | rs878481 | 2 | 233285872 | G | C | 0.408 | -0.319 (-0.401, -0.237) | 5.93E-14 | -0.182 (-0.313, -0.052) | 0.006 | N |
| cg21566642* | 2 | 233284661 | ALPPL2 | rs3748971 | 2 | 233250683 | T | C | 0.074 | -0.593 (-0.743, -0.443) | 2.68E-14 | 0.111 (-0.115, 0.338) | 0.335 | N |
| cg01901332* | 11 | 75031054 | ARRB1 | rs7927381 | 11 | 67346743 | T | C | 0.082 | 0.382 (0.247, 0.517) | 3.94E-08 | -0.191 (-0.4, 0.018) | 0.073 | Y |
| cg01901332* | 11 | 75031054 | ARRB1 | rs13087163 | 3 | 77329538 | A | C | 0.39 | -0.194 (-0.263, -0.124) | 5.82E-08 | 0.11 (-0.019, 0.239) | 0.094 | Y |
| cg08709672* | 1 | 206224334 | AVPR1B | rs77433148 | 5 | 135967502 | T | A | 0.018 | -0.804 (-1.07, -0.535) | 6.35E-09 | -0.221 (-0.784, 0.342) | 0.442 | Y |
| cg25305703 | 8 | 128378218 | CASC21 | rs9643220 | 8 | 128386926 | A | G | 0.227 | 0.343 (0.247, 0.440) | 7.09E-12 | 0.232 (0.078, 0.385) | 0.003 | N |
| cg03636183 | 19 | 17000585 | F2RL3 | rs734568 | 19 | 17015685 | T | C | 0.361 | 0.284 (0.199, 0.368) | 6.74E-11 | 0.203 (0.074, 0.332) | 0.002 | N |
| cg09935388 | 1 | 92947588 | GFI1 | rs17518433 | 1 | 92599172 | A | T | 0.236 | -0.330 (-0.421, -0.240) | 1.80E-12 | -0.186 (-0.339, -0.033) | 0.017 | N |
| cg26963277 | 11 | 2722407 | KCNQ1 | rs463924 | 11 | 2717680 | T | C | 0.304 | -0.394 (-0.482, -0.307) | 6.80E-18 | -0.277 (-0.41, -0.145) | 3.96E-05 | N |
| cg11660018 | 11 | 86510915 | PRSS23 | rs1939110 | 11 | 86515072 | T | C | 0.286 | -0.404 (-0.498, -0.309) | 2.61E-16 | -0.229 (-0.385, -0.073) | 0.004 | N |
| cg23771366 | 11 | 86510998 | PRSS23 | rs72967500 | 11 | 86505120 | T | C | 0.132 | -0.628 (-0.750, -0.506) | 1.27E-22 | -0.35 (-0.534, -0.166) | 1.93E-04 | N |

*SNP used as an instrumental variable was not replicated in the independent dataset (NSHDS)

Trans = trans mQTL (Yes/No)

Chr = chromosome

Pos = position

MAF = minor allele frequency

A1 = effect allele

**Table S4. Full results for MR of DNA methylation of 14 CpG sites on lung cancer**

| **Gene region** | **CpG** | **IV number** | **Outcome** | **OR (95% CI)** | **P value** |
| --- | --- | --- | --- | --- | --- |
| AGAP2 | cg16823042 | 1 | Lung cancer | 0.937 (0.858, 1.02) | 0.1492 |
| AGAP2 | cg23387569 | 1 | Lung cancer | 0.943 (0.871, 1.02) | 0.1492 |
| AHRR | cg05575921* | 2 | Lung cancer | 0.936 (0.870, 1.01) | 0.0809 |
| ALPPL2 | cg27241845 | 1 | Lung cancer | 0.981 (0.926, 1.04) | 0.5216 |
| ALPPL2 | cg05951221 | 1 | Lung cancer | 1.02 (0.949, 1.10) | 0.5584 |
| ALPPL2 | cg21566642* | 1 | Lung cancer | 0.922 (0.847, 1.00) | 0.0578 |
| ARRB1 | cg01901332* | 2 | Lung cancer | 0.943 (0.871, 1.02) | 0.1457 |
| AVPR1B | cg08709672* | 1 | Lung cancer | 1.08 (0.954, 1.21) | 0.2353 |
| CASC21 | cg25305703 | 1 | Lung cancer | 1.00 (0.924, 1.09) | 0.9555 |
| F2RL3 | cg03636183 | 1 | Lung cancer | 0.942 (0.864, 1.03) | 0.1723 |
| GFI1 | cg09935388 | 1 | Lung cancer | 1.03 (0.941, 1.12) | 0.5536 |
| KCNQ1 | cg26963277 | 1 | Lung cancer | 0.962 (0.903, 1.03) | 0.2364 |
| PRSS23 | cg11660018 | 1 | Lung cancer | 0.972 (0.912, 1.04) | 0.3718 |
| PRSS23 | cg23771366 | 1 | Lung cancer | 0.953 (0.901, 1.01) | 0.0864 |

IV number = the number of instrumental variables that proxied for methylation at a CpG site

* = Instrumental variables for that CpG site did not replicate in an independent dataset (NSHDS)

Where IV number = 1, the Wald ratio estimate is used.

Where IV number > 1, the Wald ratio estimates were meta-analyzed and the estimates were weighted by the inverse variance of the association with the outcome

**Table S5. The association between mQTLs and their CpG sites across the five timepoints in ARIES**

|  |  |  | **During pregnancy (28.7 years)** | | | **Middle age (46.9 years)** | | | **Birth** | | | **Childhood (7.5 years)** | | | **Adolescence (17.1 years)** | | |
| --- | --- | --- | --- | --- | --- | --- | --- | --- | --- | --- | --- | --- | --- | --- | --- | --- | --- |
| **CpG** | **Gene region** | **SNP** | **Beta** | **SE** | **P** | **Beta** | **SE** | **P** | **Beta** | **SE** | **P** | **Beta** | **SE** | **P** | **Beta** | **SE** | **P** |
| cg01901332 | ARRB1 | rs13087163 | -0.007 | 0.003 | 4.11E-02 | -0.004 | 0.003 | 2.47E-01 | -0.003 | 0.002 | 1.08E-01 | -0.003 | 0.003 | 1.73E-01 | **-0.015** | **0.003** | **2.95E-07** |
| cg01901332 | ARRB1 | rs7927381 | -0.003 | 0.006 | 5.91E-01 | -0.005 | 0.006 | 4.21E-01 | **0.015** | **0.003** | **7.89E-07** | 0.000 | 0.004 | 9.82E-01 | 0.004 | 0.005 | 4.48E-01 |
| cg03636183 | F2RL3 | rs734568 | **0.029** | **0.006** | **3.58E-07** | **0.027** | **0.005** | **4.67E-07** | **0.039** | **0.007** | **3.94E-08** | **0.031** | **0.005** | **2.12E-10** | **0.031** | **0.005** | **3.65E-10** |
| cg05575921 | AHRR | rs11744553 | 0.002 | 0.004 | 5.95E-01 | -0.005 | 0.004 | 2.05E-01 | **0.008** | **0.003** | **2.27E-03** | **0.009** | **0.002** | **1.26E-07** | 0.003 | 0.002 | 2.06E-01 |
| cg05575921 | AHRR | rs11746538 | -0.013 | 0.006 | 2.95E-02 | -0.011 | 0.005 | 3.74E-02 | **-0.014** | **0.004** | **3.13E-04** | **-0.016** | **0.002** | **4.84E-11** | **-0.010** | **0.003** | **9.57E-04** |
| cg05951221 | ALPPL2 | rs878481 | **-0.006** | **0.001** | **1.34E-06** | **-0.006** | **0.001** | **4.61E-06** | **-0.001** | **0.000** | **1.59E-03** | **-0.005** | **0.001** | **1.28E-08** | **-0.005** | **0.001** | **1.86E-12** |
| cg08709672 | AVPR1B | rs77433148 | -0.014 | 0.008 | 9.15E-02 | -0.004 | 0.008 | 6.18E-01 | 0.010 | 0.008 | 2.30E-01 | **-0.032** | **0.006** | **6.89E-07** | 0.002 | 0.007 | 8.23E-01 |
| cg09935388 | GFI1 | rs17518433 | **-0.037** | **0.009** | **3.59E-05** | -0.024 | 0.010 | 1.56E-02 | **-0.028** | **0.010** | **6.03E-03** | **-0.054** | **0.010** | **4.24E-08** | **-0.063** | **0.010** | **9.93E-11** |
| cg11660018 | PRSS23 | rs1939110 | **-0.017** | **0.003** | **6.52E-10** | **-0.014** | **0.003** | **1.24E-05** | -0.004 | 0.003 | 1.93E-01 | **-0.014** | **0.003** | **8.01E-07** | **-0.012** | **0.003** | **1.03E-05** |
| cg16823042 | AGAP2 | rs1048691 | **0.016** | **0.003** | **5.08E-08** | **0.013** | **0.003** | **1.00E-05** | **0.011** | **0.004** | **3.54E-03** | **0.019** | **0.003** | **1.30E-08** | **0.015** | **0.004** | **3.52E-05** |
| cg21566642 | ALPPL2 | rs3748971 | -0.009 | 0.006 | 1.57E-01 | -0.011 | 0.006 | 6.87E-02 | **-0.007** | **0.003** | **6.15E-03** | **-0.034** | **0.004** | **9.24E-15** | **-0.024** | **0.004** | **2.36E-08** |
| cg23387569 | AGAP2 | rs1048691 | **0.026** | **0.004** | **1.60E-10** | **0.020** | **0.004** | **2.22E-07** | **0.014** | **0.005** | **2.25E-03** | **0.027** | **0.004** | **2.62E-10** | **0.020** | **0.004** | **1.48E-06** |
| cg23771366 | PRSS23 | rs72967500 | **-0.010** | **0.002** | **2.45E-06** | **-0.014** | **0.003** | **6.74E-07** | **-0.010** | **0.001** | **1.83E-16** | **-0.011** | **0.001** | **2.08E-13** | **-0.012** | **0.002** | **1.33E-12** |
| cg25305703 | CASC21 | rs9643220 | **0.028** | **0.004** | **1.50E-10** | **0.023** | **0.004** | **1.61E-07** | **0.032** | **0.005** | **2.64E-09** | **0.020** | **0.003** | **1.06E-08** | **0.018** | **0.004** | **3.31E-06** |
| cg26963277 | KCNQ1 | rs463924 | **-0.018** | **0.002** | **1.56E-14** | **-0.015** | **0.003** | **1.22E-08** | **-0.008** | **0.003** | **5.62E-03** | **-0.012** | **0.002** | **6.33E-09** | **-0.015** | **0.002** | **7.50E-12** |
| cg27241845 | ALPPL2 | rs56080708 | **0.038** | **0.011** | **4.46E-04** | **0.052** | **0.012** | **2.50E-05** | **0.095** | **0.010** | **6.41E-22** | **0.102** | **0.010** | **8.42E-25** | **0.051** | **0.011** | **4.42E-06** |

Mean age at each timepoint is in brackets next to that timepoint header

SE = standard error, P = p value, N = sample size

Those estimates in bold are at FDR < 0.05, and so are said to have replicated in that timepoint

**Table S6. Estimates of heterogeneity of Mendelian randomization estimates across multiple SNPs**

| **CpG** | **outcome** | **N SNP** | **Q** | **P** |
| --- | --- | --- | --- | --- |
| cg05575921 | Lung cancer (ever) | 2 | 1.838 | 0.175 |
| cg05575921 | Small cell lung cancer | 2 | 0.019 | 0.890 |
| cg05575921 | Lung cancer (never) | 2 | 1.424 | 0.233 |
| cg05575921 | Lung adenocarcinoma | 2 | 0.437 | 0.509 |
| cg05575921 | Lung cancer | 2 | 0.003 | 0.960 |
| cg05575921 | Squamous cell lung cancer | 2 | 1.168 | 0.280 |
| cg01901332 | Lung cancer (ever) | 2 | 0.085 | 0.770 |
| cg01901332 | Small cell lung cancer | 2 | 0.004 | 0.953 |
| cg01901332 | Lung cancer (never) | 2 | 0.321 | 0.571 |
| cg01901332 | Lung adenocarcinoma | 2 | 0.780 | 0.377 |
| cg01901332 | Lung cancer | 2 | 0.965 | 0.326 |
| cg01901332 | Squamous cell lung cancer | 2 | 1.266 | 0.261 |

N SNP = number of SNPs used in the analysis as instrumental variables

Q = Cochrane’s Q statistic

Where P < 0.05, there is good evidence of heterogeneity across individual SNPs

**Table S7. Association of *AHRR* methylation and methylation allele score with confounding factors in the Copenhagen City Heart Study (CCHS)**

|  |  |  |  | **Methylation (%)** | | | **Allele score *** | | |
| --- | --- | --- | --- | --- | --- | --- | --- | --- | --- |
| **Confounder** |  |  | **N** | **Beta** | **95% CI** | **P** | **Beta** | **95% CI** | **P** |
| Sex | F |  | 4833 | (ref) |  |  |  |  |  |
|  | M |  | 3947 | -0.62 | -0.94; -0.29 | 2.0E-04 | -0.01 | -0.04; 0.06 | 0.72 |
| Alcohol |  |  | 8780 | 0.000 | -0.001; 0.009 | 0.85 | 0.000 | -0.000; 0.000 | 0.15 |
|  |  |  |  |  |  |  |  |  |  |
| Smoking status |  | Never | 1951 | (ref) |  |  |  |  |  |
|  |  | Former | 2559 | -3.07 | -3.54; -2.61 | 2.0E-38 | 0.004 | -0.07; 0.08 | 0.91 |
|  |  | Current | 4270 | -10.76 | -11.30; -10.22 | <1.0E-50 | 0.01 | -0.08; 0.10 | 0.82 |
| Exposure to dust |  |  | 1633 | -0.65 | -1.04; -0.26 | 1.0E-03 | -0.05 | -0.12; 0.01 | 0.11 |
| Exposure to passive smoking |  |  | 3165 | -0.46 | -0.78; -0.15 | 4.0E-03 | -0.03 | -0.08; 0.03 | 0.34 |
| Current use of tobacco to per cigarette equivalent |  |  | 8780 | -0.06 | -0.08; -0.03 | 9.0E-05 | -0.001 | -0.005; 0.004 | 0.72 |
| Cumulative use of tobacco to per pack-year |  |  | 8755 | -0.05 | -0.06; -0.04 | 4.0E-28 | -0.0005 | -0.002; 0.001 | 0.54 |

*Genotypic effects were scaled to equate to the same magnitude of effect as a per 1% increase in methylation. Regressions were adjusted for the other factors in the tab

**Table S8. One-sample Mendelian randomization analysis of the effect of AHRR methylation (%) on lung cancer risk in the Copenhagen City Heart Study (CCHS)**

|  | **Instrumental variable regression using predicted *AHRR* methylation (%)** | | | |
| --- | --- | --- | --- | --- |
|  | **Total N** | **N events** | **Age and sex adjusted HR (95% CI)** | **P** |
| **Lung cancer** |  |  |  |  |
| Total | 8758 | 357 | 0.88 (0.78; 1.00) | 0.05 |
| **Stratified by smoking status** |  |  |  |  |
| Current | 4262 | 305 | 0.90 (0.79; 1.03) | 0.12 |
| Former | 2548 | 43 | 0.86 (0.61; 1.22) | 0.41 |
| Never | 1948 | 9 | 0.83 (0.38; 1.85) | 0.66 |

HR = hazard ratio

**Table S9. Two sample Mendelian randomization analysis for *AHRR***

| **Lung cancer** | **Number of SNPs** | **Fixed effects meta-analysis** | | **Correction for correlation** | | **Heterogeneity statistics** | | |
| --- | --- | --- | --- | --- | --- | --- | --- | --- |
|  |  | **Beta** | **SE** | **Beta** | **SE** | **Q** | **DF** | **P** |
| All | 4 | -0.005 | 0.010 | -0.004 | 0.009 | 2.73 | 3 | 0.43 |
| Squamous cell | 4 | 0.003 | 0.024 | 0.004 | 0.014 | 6.65 | 3 | 0.08 |
| Adenocarcinoma | 4 | -0.022 | 0.011 | -0.022 | 0.012 | 1.06 | 3 | 0.79 |
| Small cell carcinoma | 4 | 0.002 | 0.021 | 0.001 | 0.022 | 0.14 | 3 | 0.99 |
| Never smoker | 4 | -0.003 | 0.025 | -0.003 | 0.026 | 1.37 | 3 | 0.71 |
| Ever smoker | 4 | -0.017 | 0.010 | -0.016 | 0.011 | 1.63 | 3 | 0.65 |

DF = degrees of freedom

**Table S10. Comparison of MR results with tumour-healthy tissue differential methylation**

|  |  | **Adenocarcinoma** | | | | **Squamous cell carcinoma** | | | |
| --- | --- | --- | --- | --- | --- | --- | --- | --- | --- |
|  |  | **MR** | | **Tumour/healthy tissue comparison** | | **MR** | | **Tumour/healthy tissue comparison** | |
| **CpG** | **Gene** | **Direction** | **P** | **Direction** | **P** | **Direction** | **P** | **Direction** | **P** |
| cg23387569 | AGAP2 | neg | 6.00E-03 | pos | 8.77E-01 | pos | 3.64E-01 | neg | 5.00E-01 |
| cg05575921 | AHRR | neg | 8.90E-02 | neg | 9.63E-01 | neg | 5.20E-02 | pos | 3.50E-02 |
| cg05951221 | ALPPL2 | pos | 9.16E-01 | pos | 8.00E-03 | pos | 2.73E-01 | pos | 1.00E-09 |
| cg21566642 | ALPPL2 | neg | 6.73E-01 | pos | 1.00E-04 | neg | 1.60E-02 | pos | 8.00E-07 |
| cg27241845 | ALPPL2 | pos | 5.32E-01 | neg | 7.60E-02 | neg | 1.01E-01 | pos | 2.81E-01 |
| cg01901332 | ARRB1 | neg | 4.50E-02 | pos | 3.00E-16 | neg | 7.78E-01 | pos | 8.00E-12 |
| cg08709672 | AVPR1B | pos | 1.29E-01 | pos | 3.00E-02 | neg | 8.62E-01 | pos | 2.00E-02 |
| cg25305703 | CASC21 | pos | 1.70E-01 | neg | 3.00E-05 | neg | 7.92E-01 | neg | 8.00E-04 |
| cg03636183 | F2RL3 | neg | 1.51E-01 | pos | 8.00E-04 | pos | 7.58E-01 | neg | 7.63E-01 |
| cg09935388 | GFI1 | neg | 8.31E-01 | pos | 2.00E-04 | pos | 5.67E-01 | pos | 2.00E-20 |
| cg26963277 | KCNQ1 | neg | 2.99E-01 | pos | 3.60E-02 | neg | 3.14E-01 | pos | 3.00E-03 |
| cg23771366 | PRSS23 | pos | 8.19E-01 | neg | 4.00E-09 | neg | 4.70E-02 | neg | 3.30E-02 |
| cg11660018 | PRSS23 | equal | 9.99E-01 | neg | 3.00E-08 | neg | 6.20E-02 | neg | 1.28E-01 |

pos = hypermethylated

neg = hypomethylated

For tumour/healthy tissue comparison, pos = hypermethylation of the CpG within the tumour tissue (neg is the opposite)

**Table S11. mQTL-gene expression analysis in lung and whole blood using data from GTEx**

|  |  |  |  |  |  |  |  |  | **Lung** | | | **Whole blood** | | |
| --- | --- | --- | --- | --- | --- | --- | --- | --- | --- | --- | --- | --- | --- | --- |
| **Gene** | **SNP** | **CpG** | **Trans** | **SNP pos** | **SNP Chr** | **A1** | **A2** | **MAF** | **Beta** | **SE** | **P** | **Beta** | **SE** | **P** |
| AGAP2 | rs1048691 | cg16823042 | N | 58152948 | 12 | T | C | 0.207 | 0.011 | 0.046 | 8.15E-01 | 0.053 | 0.037 | 0.153 |
| AGAP2 | rs1048691 | cg23387569 | N | 58152948 | 12 | T | C | 0.208 | 0.011 | 0.046 | 8.15E-01 | 0.053 | 0.037 | 0.153 |
| AHRR | rs11746538 | cg05575921 | N | 26366 | 5 | A | C | 0.121 | -0.009 | 0.075 | 9.01E-01 | -0.050 | 0.072 | 0.491 |
| AHRR | rs11744553 | cg05575921 | N | 427466 | 5 | C | G | 0.311 | -0.259 | 0.103 | 1.23E-02 | -0.032 | 0.110 | 0.773 |
| ALPPL2 | rs56080708 | cg27241845 | N | 233250683 | 2 | A | C | 0.078 | 0.020 | 0.131 | 8.78E-01 |  |  |  |
| ALPPL2 | rs878481 | cg05951221 | N | 233274475 | 2 | G | C | 0.408 | 0.396 | 0.123 | 1.48E-03 |  |  |  |
| ALPPL2 | rs3748971 | cg21566642 | N | 233285872 | 2 | T | C | 0.074 | 0.257 | 0.065 | 1.05E-04 |  |  |  |
| F2RL3 | rs734568 | cg03636183 | N | 17015685 | 19 | T | C | 0.361 | 0.071 | 0.048 | 1.45E-01 | -0.093 | 0.039 | 0.017 |
| GFI1 | rs17518433 | cg09935388 | N | 92599172 | 1 | A | T | 0.236 | 0.114 | 0.052 | 3.13E-02 | -0.001 | 0.036 | 0.986 |
| KCNQ1 | rs463924 | cg26963277 | N | 2717680 | 11 | T | C | 0.304 | 0.008 | 0.038 | 8.30E-01 | 0.003 | 0.028 | 0.919 |
| PRSS23 | rs1939110 | cg11660018 | N | 86505120 | 11 | T | C | 0.286 | -0.151 | 0.092 | 1.01E-01 | 0.026 | 0.042 | 0.534 |
| PRSS23 | rs72967500 | cg23771366 | N | 86515072 | 11 | T | C | 0.132 | -0.061 | 0.067 | 3.63E-01 | 0.014 | 0.027 | 0.614 |

Trans = Trans mQTL (Yes/No)

MAF = minor allele frequency

pos = position

chr = chromosome

A1 = effect allele

A2 = other allele

# Supplementary Figures

**Figure S1. Comparison of two-sample Mendelian randomization results when using the discovery (ARIES, n = 1,018) and replication (NSHDS, n = 468)**

On the left-hand side of each column the bracketed numbers represent the number of instrumental variables for that CpG site.

**Figure S2. DNA methylation – lung cancer Mendelian randomization effect estimates in ever and never smokers**

On the left-hand side of each column the bracketed numbers represent the number of instrumental variables for that CpG site. * indicates that the SNP(s) being used to instrument that CpG site are more than 1MB away from the CpG site in the genome (trans).

**Figure S3. Mendelian randomization of DNA methylation on three lung cancer subgroups**

On the left-hand side of each column the bracketed numbers represent the number of instrumental variables for that CpG site. * indicates that the SNP(s) being used to instrument that CpG site are more than 1MB away from the CpG site in the genome (trans). Squamous = squamous cell carcinoma, Small = small cell carcinoma, Adeno = adenocarcinoma

**Figure S4. Associations of mQTLs and smoking behaviours**

#

Some SNPs that were genotyped in the TRICL consortium were not within the TAG consortium, thus were not available for analysis here. Units for the outcomes: age of smoking initiation = log years, ever vs. never smoked = log odds, former vs current smoker = log odds.

**Figure S5. Two-sample Mendelian randomization analysis of DNA methylation at *AHRR* on lung cancer**

A

B

C

D

E

F

Analysis are divided into these categories: a) All lung cancer b) Squamous cell carcinoma c) Adenocarcinoma d) Small cell carcinoma e) All lung cancer in never smokers only f) All lung cancer in ever smokers only
